# Mate-Pair Sequencing as a Powerful Clinical Tool for the Characterization of Cancers with a DNA Viral Etiology

**DOI:** 10.3390/v7082831

**Published:** 2015-08-07

**Authors:** Ge Gao, David I. Smith

**Affiliations:** Division of Experimental Pathology, Mayo Clinic, Rochester, MN 55905, USA; E-Mail: gao.ge@mayo.edu

**Keywords:** DNA virus, human papillomavirus, cervical cancer, oropharyngeal cancer, mate pair sequencing

## Abstract

DNA viruses are known to be associated with a variety of different cancers. Human papillomaviruses (HPV) are a family of viruses and several of its sub-types are classified as high-risk HPVs as they are found to be associated with the development of a number of different cancers. Almost all cervical cancers appear to be driven by HPV infection and HPV is also found in most cancers of the anus and at least half the cancers of the vulva, penis and vagina, and increasingly found in one sub-type of head and neck cancers namely oropharyngeal squamous cell carcinoma. Our understanding of HPVs role in cancer development comes from extensive studies done on cervical cancer and it has just been assumed that HPV plays an identical role in the development of all other cancers arising in the presence of HPV sequences, although this has not been proven. Most invasive cervical cancers have the HPV genome integrated into one or more sites within the human genome. One powerful tool to examine all the sites of HPV integration in a cancer but that also provides a comprehensive view of genomic alterations in that cancer is the use of next generation sequencing of mate-pair libraries produced from the DNA isolated. We will describe how this powerful technology can provide important information about the genomic organization within an individual cancer genome, and how this has demonstrated that HPVs role in oropharyngeal squamous cell carcinoma is distinct from that in cervical cancer. We will also describe why the sequencing of mate-pair libraries could be a powerful clinical tool for the management of patients with a DNA viral etiology and how this could quickly transform the care of these patients.

## 1. Introduction

DNA viruses are known to play very important roles contributing to the development of a number of different cancers and it has been estimated that 15% of all human cancers may be attributed to viral infections [1]. There are several DNA viruses that are involved in the development of human cancers. Epstein-Barr virus (EBV), a large double strand DNA virus (172 kb) has been shown to be associated with B and T cell lymphomas, Hodgkin’s diseases and nasopharyngeal carcinoma [2,3,4,5]. Persistent infection of specific strains of Human Papilloma Virus (HPV) is known to be directly involved with the development of cervical cancer, several other anogenital cancers as well as sub-types of head and neck cancer [6,7]. Hepatitis B virus (HBV) infection is known as a significant global health problem with an estimated two billion people infected and 1.2 million deaths per year due to HBV leading to the development of hepatitis, cirrhosis and ultimately hepatocellular carcinoma [8]. 

The different mechanisms whereby these DNA viruses are responsible for the development of different cancers have not been fully elucidated. However, considerably more is known about how HPV is involved in the development of cervical cancer. HPV infection into cervical epithelium results in cellular immortalization but not transformation, thus, additional alterations are required for the development of invasive cancer [9,10,11]. Invasive cervical cancers almost always have HPV integrated into the human genome and this appears to trigger genome-wide genomic instability which most likely helps to provide the necessary additional alterations that lead to tumor progression [12]. Similarly, in 85%–90% of HBV-related hepatocellular carcinomas (HCCs) the HBV genome is found to be integrated into the human genome but at multiple sites [13]. However, since both HPV and HBV are found integrated at multiple positions within the human genome in different cancers it was first assumed that the sites of viral integration were random and unimportant. More detailed studies have revealed that both HPV and HBV integrations are not random and that genes at or near integration sites could be playing an important role in the eventual cancers that develop.

The detection of viral integration sites in the host genome has been a challenge. The traditional methods used for detecting virus integrations are always labor extensive and suffer from a lack of accuracy and sensitivity. With the dramatic increases in sequence output provided by next generation sequencing it is now possible to identify and characterize most viral integrations and the resulting changes at and around integration sites with great specificity. In this review, we will mainly focus on the discussion of HPV infection and integrations in the development of cervical cancer and one sub-type of head and neck cancers, oropharyngeal squamous cell carcinomas (OPSCCs). This will serve as an example that virus integration events including the sites of the virus integration both within the virus and also the sites of integration in the human genome actually play very important roles in cancer development. Indeed, the information revealed by virus integration also has been reported as being related to the diseases outcome. We will discuss how one specific application of next generation sequencing based upon paired end sequencing of mate-pair libraries offers a powerful tool for the characterization of the HPV-driven OPSCCs. In addition we will describe how this could be a very powerful clinical tool which could transform management of patients with all cancers with a DNA viral etiology.

## 2. HPV and Cervical Cancer

Cervical Cancer is the second most common cancer worldwide and it has been estimated that it is responsible for 600,000 incident cases and 300,000 deaths each year [14]. Persistent infection of high risk sub-types of human papillomaviruses (HPV) is the primary cause of the development of cervical cancer and they are known to be involved in the pathogenesis of over 99% of cervical cancers [15,16]. HPV is a small, non-enveloped double stranded DNA virus that often infects mucosal and cutaneous epithelial cells. There are over 120 HPV types that have been characterized and they are designated as high risk and low risk based on whether or not they are associated with an increased risk of cancer [17]. Low risk HPV sub-types are mainly responsible for the development of papillomas (warts) [18], but infection with high risk HPV sub-types can lead to the development of cervical and several other cancers [19]. The viral genome is around 8 Kb in size and is amplified initially as episomal elements [20]. Women whose immune system does not clear the virus are at an increased risk of developing cervical cancer and in these women long-term infection can sometimes lead to the viral genome being integrated into the host genome. The integration of HPV DNA into the host cell genome has been indicated as a key event for the malignant progression of cervical carcinoma. In vitro studies have suggested that cells with integrated HPV possess a selective growth advantage compared to cells that maintain just episomal copies of the viral genome [21]. The viral integrations in cervical cancers are observed in 100% of HPV 18 positive cases and over 80% of the HPV 16 positive cases [22,23]. HPV integrations are also correlated with cervical intraepithelial neoplasia (CIN) grades and overall disease progression [24,25]. Although a small percentage of cervical cancers do develop while the HPV-DNA remains in just the episomal form, it has been shown that in these cases there is a high episomal HPV copy number which is accompanied by increased viral oncogene expression [26]. 

Studies in cervical cancer have shown that the viral early genes E1 and E2 were the preferential sites for integration and the E2 region was found to be more commonly disrupted or deleted than any other sites [27]. The E2 protein negatively regulates the expression of the viral oncogenes E6 and E7 [28], thus loss of the E2 ORF during integration results in increased E6 and E7 mRNA transcription and this leads to increased oncoprotein expression. The E6 protein encoded by high risk type HPVs stimulates the degradation of the tumor suppressor p53 [29,30] and the E7 protein binds strongly to retinoblastoma (*pRB*) [31], thus, the overexpression of these two proteins results in the inhibition of the functions of these two important tumor suppressors. This causes an increase in genomic instability thus ultimately leading to malignant progression [32,33]. Initial studies which examined the sites of HPV integration into the human genome in different cervical cancers found integrations throughout the genome thus it was just assumed that the sites of HPV integration in the human genome were random and played no role in the eventual cancers that developed, and thus the key event in HPV integrations was the disruption of the E2 gene and the activation of expression of the E6 and E7 oncogenes.

There are different approaches that can be used to determine the physical status of HPV in an individual cervical cancer. One indirect approach is to measure the expression of the E2 transcript by real time PCR assuming that a unique region of the E2 open reading frame (ORF) is most often deleted during HPV 16 integration. Hence, by measuring the expression of the E2 transcripts and comparing it to the expression of the E6 or E7 transcripts, using real-time RT-PCR it has been suggested that the virus was integrated if E6/E7 expression was high, but E2 expression was low [34]. However, this expression based assay alone cannot distinguish the integrated forms in the presence of episomal HPV. *In situ* hybridization is another common technique used to determine the physical status of HPV by characterizing if there is hybridization to just a single position in interphase nuclei or at multiple positions across the nucleus. The observation of diffuse signals would indicate the presence of copies of episomal HPV while the observation of punctate nuclear signals would suggest that HPV is integrated into the cell genome [35]. However, both of these techniques do not provide information about the precise sites of viral integration. In addition, they frequently provide inaccurate information as to whether the virus is integrated or not.

There are other techniques that have been utilized that are able to characterize the sites of HPV integration both within the HPV genome and the precise integration sites in the human genome. These include Amplification of Papillomavirus Oncogene Transcripts (APOT) [36], Restriction Site Oligonucleotide PCR (RSO-PCR) [37], and Detection of Integrated Papillomavirus Sequences (DIPS) [38]. A number of years ago, we utilized RSO-PCR to characterize the sites of HPV16 and HPV18 integrations in cervical cancer. RSO-PCR is a powerful technique but it requires that the HPV integration site occurs close to one of the restriction sites utilized for RSO-PCR, and that the integration site itself is not occurring within highly repetitive DNA sequences. Although HPV integrations were found throughout the human genome, our studies revealed that 50% of the HPV 16 integrations occurred within one of the highly unstable common fragile site (CFS) regions [39]. HPV18 integrations were found to also frequently occur within CFS regions (60% of the time) and we detected a hot-spot for integrations around the *c-myc* oncogene [40]. These results suggest that the sites of HPV integration in the human genome may not be random. Another reason for the frequent integration at or near CFS regions is that the papillomavirus genomes associate with BRD4 to replicate at these sites in the host genome [41]. However, it would still be interesting to know whether these integration events are occurring simply because of the instability within the highly unstable CFS regions, or if there are genes at or near these integration sites that could be playing important roles in the eventual cervical cancers that develop.

## 3. HPV and Other Anogenital Cancers

HPV is also known to be associated with a spectrum of other anogenital cancers. It has been estimated that HPV is involved in over 80% of anal cancers and approximately 40% to 50% of penile, vulvar and vaginal cancers [42]. Persistent infection with high-risk sub-types of HPV is considered to be responsible for a large proportion of anal cancers and the incidence has been reported to be increasing in the USA, as well as in European countries [43,44]. It has also been shown that there is a strong relationship between developing anal cancer and the development of other genital cancers [45,46].

While HPV is found to be involved in the development of other anogenital cancers, not many studies have been done to investigate the physical status or the sites of HPV integrations in these cancers. Limited research examining vulva intraepithelial neoplasia and vaginal cancer by DIPS and APOT have suggested that HPV integrations are indeed common in these cancers and that there is active transcription of the HPV genome. Preliminary studies have also suggested that HPV integration frequently occurred within the common fragile sites [47,48]. However, considerably more work needs to be done in the non-cervical anogenital cancers to determine whether HPV plays an identical role in the development of these cancers as it does in cervical cancer.

## 4. HPV and OPSCC

In the United States over the past two decades, there have been dramatic increases in the incidence in OPSCC although the overall incidence of other sub-types of head and neck cancer have been dropping due to decreases in tobacco consumption. OPSCC is a subtype of Head and Neck cancer derived from the tonsil and the base of the tongue, as well as the cancers from the walls of the pharynx and the soft palate [49]. It has been shown that an increased incidence of HPV infection, especially that from HPV16, was the key factor attributing to this dramatic increase. In 2007, the International Agency for Research against Cancer (IRAC) acknowledged HPV, in addition to smoking and alcohol consumption, as the key risk factors for the development of OPSCC.

At the Mayo Clinic over the past several decades the frequency of OPSCCs that are HPV positive has increased from less than 20% to over 80%. However, there is far greater complexity in the characterization of viral-driven OPSCCs as compared to similar studies in cervical cancer. The first is that many OPSCC patients have a history of smoking and drinking. How these risk factors interact with HPV to cause many OPSCCs is at present unclear. Recent studies have demonstrated that smokers are much more likely to be HPV-positive [50], but whether this is due to smokers being more likely to engage in high-risk behaviors or whether there is an interaction between smoking and HPV infection and/or integration is unclear. 

A greater complexity is the fact that not all OPSCCs that contain HPV sequences are the same. Some patients contain HPV sequences but very low expression of HPV-specific transcripts. These are thought to represent “latent” HPV infections [51]. Whether or not the viral sequences present in the cancers in these patients helped to contribute to the development of that cancer is unknown. In contrast, other patients have HPV sequences present and very robust expression of HPV-specific transcripts, and these are presumed to represent “active” HPV infections [51]. 

Whether or not an OPSCC patient has HPV sequences present and active transcription of those sequences is turning out to be very important clinically and biologically. In spite of the fact that tumors in patients with HPV sequences present tend to be larger and of a later stage, patients who are HPV positive are found to have a better overall clinical outcome than those whose tumors are HPV negative [52]. This may be because more DNA damage and genomic alterations are present in the HPV-negative OPSCC tumors, which are more than likely caused by the combination of smoking and drinking. Due to these findings there is active discussion about whether or not there should be a de-escalation in therapy for those patients whose OPSCC tumors are HPV positive [53]. However, in light of the fact that some HPV positive OPSCCs have low expression of E6/E7 while others have high expression just measuring the presence of HPV DNA in patients’ tumors is insufficient. A viable alternative that is currently being explored instead of simply testing tumors for the presence of HPV sequences or measuring p16 expression (as p16 expression is increased in HPV positive cancers) is to accurately measure either E6 and E7 transcript expression or E6/E7 protein expression. For example, RNA scope is one commercially available approach to measure the expression of the E6/E7 transcripts and this is being tested as a more accurate way to determine the presence of HPV in a specific OPSCC and more importantly whether or not that OPSCC has an active or a latent infection with HPV [54].

All the work in characterizing HPVs role in the development of cervical cancer has led many to assume that this virus must play a similar or identical role in all other cancers where it is found. However, the studies in characterizing the physical status of HPV in OPSCC in a large number of these cancers are limited. Studies from two groups have examined the E2/E6 expression ratio by real-time RT-PCR and based on this assay one group determined that 48% of the cancers they tested had HPV integration while the other found that 78% of their cancers had HPV integrations [55,56]. 

Utilizing the fluorescence *in situ* hybridization assay and the detection of punctate nuclear signals, Mooren *et al.* [57] reported that 42% of OPSCCs have HPV integrated into the human genome. However, as mentioned above, there are severe limitations with both assays as neither of them are accurate enough and both cannot determine where the virus integrates.

Previously, we reported that we could not detect any HPV integration from 40 HPV16 positive OPSCC by using restriction site oligonucleotide PCR (RSO-PCR) [58], while the same method was able to identify HPV-human junctions in many cervical cancers [39,59,60]. All of these studies together suggest that HPV integrations do occur in OPSCC but that the frequency of integrations may occur less than it does in cervical cancer.

## 5. Recent Studies Detecting HPV Integrations by Next Generation Sequencing

The development of next generation sequencing based upon massively parallel sequencing has transformed our ability to characterize genome changes in cancer [61,62,63] and has also provided tools to examine in detail viral integration sites and the effect that these integrations have on the surrounding regions. Over the past several years these technologies have resulted in dramatic increases in sequence output and recently have even reduced the cost of whole human genome sequencing to less than $1,000 per genome (when just a few years ago the cost for doing this was considerably greater) [64].

Whole genome sequencing is known to be a powerful technique to identify viral integration sites and the alterations around those sites caused by the integration event. This was aptly demonstrated by the full sequence characterization of the HeLa genome, a cell line established from the cervical cancer patient, Henrietta Lacks. The whole genome sequence of the HeLa cell line revealed that this HPV18 positive cervical cancer had HPV integrated into the hot-spot region for HPV18 integrations which we previously had identified in the vicinity of the *c-myc* oncogene on chromosome 8q24.21 [65]. The HPV18 integration resulted in amplification of the HPV18 genome with some portions amplified up to 32-fold while others were amplified only eight-fold. The integration occurred 500 kb awayfromthe *c-myc* oncogene (within one of the CFSs that flank *c-myc*), and functional studies demonstrated that this integration was responsible for increased expression of *c-myc*. This is most likely the reason that the cervical cancer that was responsible for the death of Henrietta Lacks was so aggressive [65]. This demonstrated that there could be dramatic alterations in the chromosomal region surrounding an HPV integration and that human genes in the vicinity of those integrations could themselves be playing an important role in the eventual cancer that forms.

Recent studies using whole-genome sequencing analyzing cervical intraepithelial neoplasias, cervical carcinomas and cervical cancer-derived cell lines provided further support that the unstable CFS regions are indeed hot spots for HPV integrations in the human genome. Three very large CFS genes *FHIT*, *LRP1B* and *DLG2* were the sites of HPV integrations in multiple cervical cancers and the protein expression of *FHIT* and *LRP1B* was down regulated when HPV integrated in their introns [66]. *FHIT* is a known and very important tumor suppressor gene that resides within the most highly unstable CFS region in the human genome [67,68]. Both *LRP1B* and *DLG2* are very attractive tumor suppressor candidate genes [69,70,71]. These studies further support the contention that not only is HPV integration within the human genome non-random but that human genes in the vicinity of these integrations could be important. 

Whole genome sequencing is one strategy for studying HPV integrations but a number of other investigators have instead focused their efforts on just the coding portion of the genome and have therefore been doing whole exome sequencing instead (which at 38 Mbs is a much smaller fraction of the genome). This provides a much more tractable characterization of alterations within cancer genomes and solves many of the informatics and cost problems associated with whole genome sequencing. Unfortunately, this approach does not provide as comprehensive information about changes in copy number throughout the genome, nor does it provide information about HPV integration events, unless they occur within the relatively small proportion of the genome that codes for protein. We recently performed whole exome sequencing on cervical adenocarcinomas derived from Hong Kong women. These studies revealed that 13 of the 15 cancers characterized appeared to have HPV integration events near coding genes [72], but these were not PCR validated. This further suggests that the HPV integration events are not random and that there could be a selection for HPV integration near to genes and these could be playing an important role in the eventual cancers that develop.

Another alternative to whole genome sequencing for characterizing HPV integrations is to analyze whole transcriptome sequencing (called RNAseq). The advantage of this is that it not only identifies the level of transcription of human genes, but also those of the HPV genome. If the HPV integration has resulted in the production of a fused transcript it too can be detected from this data. 

Khoury *et al*. [73], analyzed the RNA seq data provided from the The Cancer Genome Atlas (TCGA) Project characterization of head and neck cancers and they were able to detect 24 tumors from 36 HPV positive squamous cell carcinomas of the head and neck that appeared to have fused HPV-human transcripts. From their findings, of the tumors with HPV integration, 18 have both HPV E6 and E7 integration sites, four have only E7 integration sites and two have only E6 integration sites. These findings provided new insights in elucidating the potential mechanisms whereby HPV integration might be involved in cancer development. However, all of their analysis was based solely on their bioinformatic algorithms that could putatively identify fused HPV human gene transcripts from the short reads and they did no direct experimental validation as they did not have access to the RNAs used for the transcriptome sequencing. Studies of RNAseq data obtained in the analysis of breast cancers revealed that only 8.5% of the putative fusion genes could be confirmed by RT-PCR from 83 putative fusion transcripts tested [74] given the fact that there was a lot of misalignment of the short reads at the junction sites. Thus, it is unclear how many of the putative integrations detected by Khoury *et al*. [73], were real, hence the proportion of HPV positive head and neck cancers from the TCGA data that have integrations is still unclear. Tang *et al.* [75] also reported the viral expression and host gene fusions detected by massively parallel sequencing in 19 cancer types. In their work, they applied a stringent procedure for detecting HPV integrations from RNA seq and validated the methodology using the whole genome sequencing data from nine HPV positive HNSCC tumors. It was found that eight of nine HPV integrations detected by RNA seq were supported from whole genome sequencing data. In their report, they observed high integration frequency for HPV18 (100%) but a lower frequency for HPV16 (58.5%). In addition, they also detected recurrent fusion events including HPV insertion in *RAD51B* and *ERBB2*.

Of all these massively parallel sequencing techniques used, the most powerful technique to characterize all HPV integrations is WGS. Exome sequencing and RNA seq will only detect a sub-set of integrations that occur near exons or cause exon-HPV transcript fusions. However, there are a number of limitations to whole genome sequencing. The first is that the full cost of whole genome sequencing is considerably greater than just the cost of generating the sequence. While the cost of WGS has dropped considerably in the past few years that is only a fraction of the total cost of generating the complete sequence of a normal or a cancer genome. An important cost is for alignment the short read sequences into a fully integrated genome sequence. In addition, it is frequently necessary to validate putative alterations detected in a cancer genome with PCR and subsequent sequencing which can greatly increase the cost of analyzing a single cancer genome. Long term storage of the sequences generated (which is something that is very important for clinical genome sequencing) can be quite expensive as well. Finally, interpreting WGS and determining which of the alterations detected are clinically important is still in its infancy.

## 6. Mate Pair Sequencing

The limitations of whole genome sequencing have precluded it from yet becoming an important clinical tool for the characterization of cancer genomes. There is, however, a powerful and much cheaper alternative to whole genome sequencing which can provide a great amount of information about genome-wide changes in viral driven cancers namely the construction of mate-pair libraries. By producing larger sized inserts and then circularizing them to capture just the ends of the large inserts one can generate libraries ready for sequencing which can then be paired-end sequenced. This then generates sequence from the two ends of the much larger original DNA fragments isolated.

**Figure 1:**
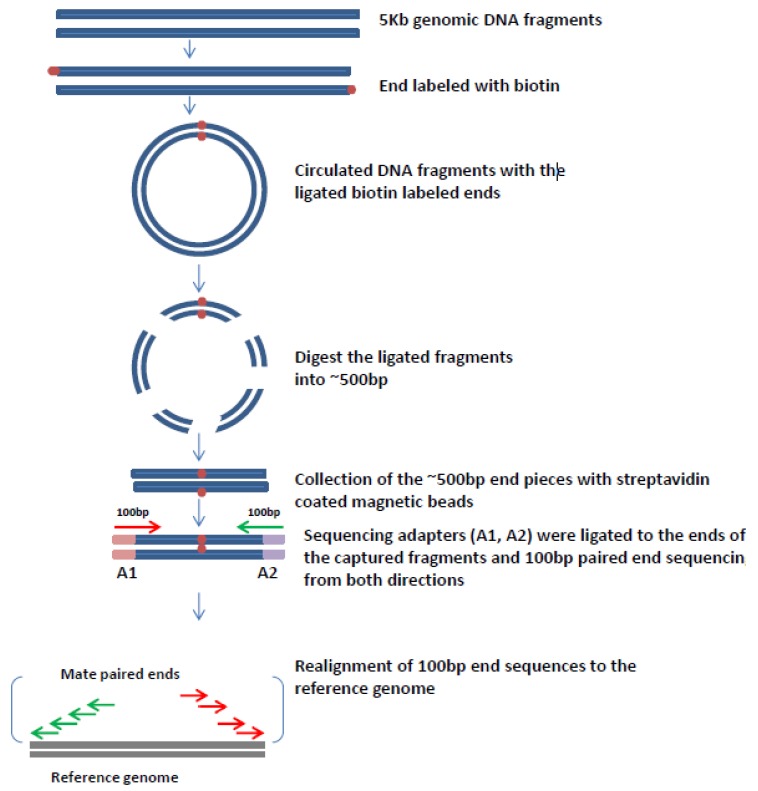
Principles of mate pair libraries construction and the bioinformatics analysis. The genome to be characterized is fragmented into pieces and then 5 kb genomic DNA fragments are isolated. These are then biotinylated and the resulting end-labeled fragments are circularized. The two fragment ends then become adjacent to each other. The circularized DNA is then fragmented into small (300–500 bp) pieces and the biotinylated fragments are collected and purified by affinity capture with streptavidin-coated magnetic beads. Sequence adapters (A1, A2) are then ligated to the ends of the captured fragments and 100 bp paired ends are sequenced from both directions. The 100 bp sequence reads are then realigned to the reference genome to determine their genome location. Although only 200 bp of sequence is generated from these mate-pair fragments they actually provide inferred information about the region between the two ends, which is referred to as bridged coverage.

Figure 1 (below) shows how mate-pair libraries are constructed. In our experiment, high throughput genomic mate pair libraries were prepared by the Mayo Clinic Genome Core Facilities using the Illumina Mate Pair protocols and their available kit (Nextera Mate Pair Sample Preparation kit, Illumina). Whole genome DNA is fragmented (either mechanically or with partial restriction digestion) to generate 5 kb or greater sized fragments. The larger fragments are then size selected and end labeled with biotin. These fragments are then circularized and then the resulting circles are fragmented to generate the two ligated biotin-labeled end fragments which are then are captured using streptavidin-coated magnetic beads. These fragments are then sequenced with paired end sequencing to generate 100 base pairs of sequence from both ends of each mate-pair. After sequencing and alignment against a reference genome the resulting 200 base pairs of sequence is actually providing sequence information about the entire region between the two ends, which is referred to as bridge coverage [76]. Thus, even if there is only 100 base pair of DNA sequencing at each end of an original mate-pair one can infer information about the entire region between those two mate-pair ends, hence a small amount of DNA sequencing actually provides considerably more information about the entire region covered by those two mate-pairs. With this strategy and the bridged coverage provided by it, one can determine genetic alterations such as small insertions and deletions occurring across a genome with much less overall sequencing. With this technique and, for example, with a 5 kilobase mate-pair library it is possible to characterize genome alterations in a cancer with just 2–5 Gbs of DNA sequencing. This does not provide individual nucleotide resolution, but will identify genome-wide changes in cancer, and will also identify most of the virus integration events that have occurred in the DNA of a viral-driven cancer.

The Biomarker Discovery Program of the Mayo Clinic Center for Individualized Medicine, under the direction of George Vasmatzis has pioneered the use of this strategy to characterize cancers. They have further developed a powerful algorithm to characterize the generated mate-pair sequence data. This is based upon a binary mapping algorithm which greatly simplifies aligning of DNA sequences. Vasmatzis and co-workers have used their algorithm and mate-pair sequencing to characterize many different cancer genomes [76,77,78] and have successfully detected structural variations, as well as fusion genes in lymphomas, prostate cancers, and breast cancer cell lines. 

Mate-pair sequencing can also provide a powerful alternative for whole genome sequencing as the longer DNA fragments can be used to bridge together DNA fragments even in the presence of highly repetitive DNA sequences which are rife in human and many other complex genomes. Companies, such as Lucigen, have been working to generate considerably longer mate-pair libraries and these have proven to be powerful tools for fully assembling the sequence derived from complex genomes [79,80]. The only limitation is that the larger the size of the DNA fragments used for mate-pair sequencing the more input DNA is required to construct these libraries.

## 7. Mate-Pair Sequencing to Characterize OPSCCs

We have used mate pair sequencing to study OPSCC and have demonstrated that this alternative approach is less costly and can serve as a very powerful tool to detect genome structural variation, HPV integration events as well as the HPV copy number with high sensitivity and specificity. In our study, we performed mate pair sequencing on DNA isolated from both tumor and adjacent normal tissues from 20 OPSCC patients, with 14 of them being HPV 16 positive and six being HPV 16 negative [81]. Over 20 different HPV genome sequences were inserted into the mapping algorithm designed by Vasmatzis [76] and each of them was assigned as an individual artificial chromosome in addition to the 23 pairs of human chromosome. The data was then analyzed with the mapping algorithm that was internally used at Mayo Clinic and we specifically focused on the detection of any putative inter-chromosomal translocations between one of the 23 human chromosomes and any of the artificial HPV sub-type chromosomes, which could represent viral-human junctions caused by HPV integration events. From 13 HPV16 positive tumors, two tumors were identified as potentially having HPV integration with one of them detecting two integration sites. One tumor which was thought to be negative for HPV sequences (but detected with HPV16-specific primers) actually had not only HPV26 sequences present within it but HPV26 integrated into one position within the human genome. All these HPV integration events were successfully validated by PCR amplification and each virus integration junction site was successfully mapped both with respect to the site of integration within the virus as well as within the human genome by Sanger sequencing. This study revealed that only 21.4% of OPSCCs had HPV integrated into the human genome which is considerably less than what is observed in cervical cancer, and also we did not observe the E2 disruption in any of the four integration events in these OPSCC tumors while E2 is quite frequently disrupted in Cervical Cancer. These results indicate that the episomal HPV could also play important roles in the development of OPSCC and certainly more investigation should be done to understand the potential mechanisms behind it.

The mate pair sequencing also detected bridged coverage of various HPV genomes present in each of the OPSCC samples, irrespective of whether or not HPV was integrated in that tumor. When the HPV bridged coverage is very high, it generally correlates very well with HPV copy number. By comparing the HPV DNA bridged coverage with the HPV E6 and E7 transcriptional oncogene expression, apart from one exception, the bridged coverage of HPV DNA sequence was consistent with the level of HPV E6 and E7 transcription in 12 of 13 samples [81]. 

Our findings suggested that HPV could be playing different roles in the development of different OPSCCs and many of these are quite distinct from its well characterized role in cervical cancer. Thus additional studies are needed to determine the different roles that HPV plays in the development of OPSCC. Most importantly, based on our results, most HPV positive patients have just episomal HPV sequences present. How having just HPV present in the episomal form and sometimes with a low copy of HPV contributes to carcinogenesis of OPSCC will need further investigation.

Although this is the first example for the virus integration detection using the mate pair approach, the principles of the artificial algorithm mapping demonstrated that this approach could be used for the detection of other DNA virus integration events in other cancers. In addition, the high sensitivity and specificity of this technique for detecting structural variations and the gene fusions which has already been demonstrated in both hematologic malignancies and solid tumors. In an analysis of peripheral T-cell lymphomas (PTCL), Vasmatzis and Feldman’s group identified 13 recurrent abnormalities including inter and intra chromosome rearrangements in which five of them occur within *P53* related genes (*TP53*, *TP63*, *CDKN2a*, *WWOX* and *ANKRD11*) and precise breakpoints were successfully confirmed by PCR and Sanger sequencing. Additional studies from 190 PTCL tissue samples revealed that 5.8% (11 out of 190) of the cases had *TP63* rearrangements and PTCL patients who carried the *TP63* rearrangements had significantly worse survival compared with patients without the *TP63* rearrangements [78]. In addition, Mate Pair sequencing also identified recurrent t(6;7)(p25.3;q32.3) translocations in ALK negative anaplastic large cell lymphomas and several novel deletions/translocations in myelofibrosis [77,82]. All these indicated that mate pair library sequencing could be used as a powerful tool for translocation discovery at the genome level. Our study on HPV integration in OPSCC further demonstrated that when the virus genomes were used as artificial chromosomes incorporating into the mapping algorithm, the virus integration events could be detected as inter-chromosome translocations between these artificial chromosomes and the true human chromosomes, thus this approach can be used to analyze viral etiology in different cancers.

In our study, in addition to HPV integration events, the data generated from mate pair sequencing also detected inter and intra chromosome translocations in the OPSCC tumor samples. We have successfully validated the chromosome translocations and the novel fusion genes were indeed present in the tumor sample but not in the matched normal samples using PCR and Sanger Sequencing (data not published). In OPSCC, the amount of genome rearrangement rate is not as high as found in other cancers, such as breast. Currently we are investigating if there is any relationship between the virus integration and the genome translocation in the OPSCC, and how these events are related to patients’ clinical outcome, as well as if tumors with greater genomic instability tend to be more likely to have viruses integrated into the genome.

Thus, from what we have observed from our study in the HPV related OPSCC, the sequencing of mate pair libraries has generated information regarding the virus integration events, copy number variations, as well as the genome translocation information. All these will provide insight not only to potential mechanisms involved in the cancer development, but also to the biomarkers discovery for disease diagnosis, prognosis and potential therapeutic targets.

## 8. Other Information Provided by Mate-Pair Sequencing

Mate-Pair sequencing is thus a powerful tool for the characterization of the physical status of HPV, or other DNA viral genomes, in cancer. Not only could it help us to determine the sites of HPV integration and how that integration disrupts the human genome at those integrations but it also provides information about the copy number of HPV in each cancer. In addition, the mate-pair sequencing also provides information about changes in copy-number throughout the genome. Thus, we could determine with very high resolution regions of chromosomal amplification as well as regions of deletion.

It is well known that cancers that have more genomic instability generally have a worse overall clinical outcome [83,84]. Thus, mate-pair sequencing could differentiate OPSCCs that have less genomic instability and would therefore expect to have a better overall prognosis. This could be a powerful clinical tool to help to stratify patients with OPSCC, as well as other cancers. Future studies with many more patients linked to their clinical data could help to identify specific chromosomal changes (distinct from the HPV integration events themselves) which could prove to be important in determining which patients have more *versus* less aggressive cancers.

Another thing that is obtained from limited sequencing of the mate-pair libraries is the identification of cancer-specific alterations. These could be HPV integration events (in those OPSCCs that do have viral integration) but could also be inter- and intra-chromosomal translocations. These could serve as powerful biomarkers for monitoring the clinical course of treatment for that cancer patient.

## 9. Digital PCR to Monitor the Clinical Course of Cancer 

The ability of limited sequencing of mate-pair libraries to identify cancer-specific alterations may actually turn out to be the most powerful information provided by this approach. It is well known that circulating tumor-free DNA can be detected in the blood of patients with all different types of cancer. The amount of this circulating DNA is also indicative of the amount of the primary tumor present for solid tumors [85]. Finally, this DNA has a relatively short half-life [86]. Hence, having a way to digitally measure the concentration of this circulating tumor-free DNA can be a powerful tool that can be used to monitor patients during their clinical treatment.

Patients with OPSCC are generally first surgically treated and then this is followed up with both chemotherapy and radiotherapy treatments. These subsequent procedures are costly and can severely compromise the quality of life for patients. It is for this reason that clinicians are looking for ways to identify less aggressive cancers which could be treated but with a de-escalation of therapies. Thus, the finding that patients whose tumors are HPV positive have a much better clinical outcome has led to the suggestion that these are precisely the patients who could have less aggressive therapies. Unfortunately, this is not a perfect strategy as many HPV positive patients do have recurrence. However, the ability to quantitatively measure circulating tumor-free DNA could be a powerful tool to determine those patients whose surgical procedure has resulted in the removal of all tumor.

In addition, having the means to determine when tumors are beginning to recur could provide a powerful tool for clinicians to monitor an individual patients’ response to therapy. The relatively non-invasive detection of the concentration of tumor-free DNA in the blood of patients could prove to be a powerful tool to monitor in real-time how patients are responding to therapies. In the case of chemotherapy this could also be used to quickly take patients off of chemotherapeutic agents that are not working and substituting others that might prove more effective.

However, the problem with the detection of circulating tumor-free DNA is complicated by the fact that the concentration of tumor DNA could be a very small fraction of the total DNA present in blood. The identification of cancer-specific alterations by mate-pair sequencing (and especially novel DNA junctions caused by either inter-chromosomal translocations or the intra-chromosomal junctions resulting from insertions or deletions on any single chromosome) provides cancer-specific markers that could be both detected and quantified.

While this could be done by various real-time PCR techniques, a more powerful tool with much greater sensitivity is the use of digital droplet PCR [87]. Various companies can produce large numbers of such very small droplets. For example, the Bio-Rad Digital PCR machine can produce tens of thousands of individual droplets. Alternatively, the Rain-Dance Digital PCR platform can produce 10 million droplets. By fractionating dilutions of DNA isolated from the blood of cancer patients in the presence of droplets with PCR primers specific for the detection of cancer-specific alterations it then becomes possible to not only detect the circulating tumor-free DNA, but to precisely digitally determine the concentration of that DNA. 

Work on a variety of different cancers has therefore begun to determine the feasibility of this approach for monitoring the clinical treatment of cancers. A recent comparison of conventional quantitative PCR and droplet digital PCR for the measurement of clinically significant EGFR mutation found that while quantitative PCR could detect samples where the mutant molecules were present at just 5% or even down to 1% , that the digital PCR method could reliably detect 0.5% and down to 0.1% mutation rates [88]. A similar study following patients after colorectal cancer surgery found that this approach was a non-invasive, exquisitely specific and highly sensitive approach for monitoring disease load, and also had the potential to provide clinically relevant lead times compared to conventional methods [89]. Cutaneous melanomas that harbor the BRAF (V600E) mutation can be treated with BRAF inhibitors. Sanmamed *et al.* [90] demonstrated that using digital droplet PCR that they could even detect the V600E mutation at a fractional abundance of 0.005% to the wild type gene. This approach has also proven useful for the detection of rare copies of the BCR-AbL1 transcript in patients with Philadelpha-positive acute lymphoblastic leukemia [91].

Therefore, this approach could be a very powerful tool to help to monitor patients with OPSCC. Mate-pair sequencing of the primary tumor, obtained at the time of surgery could identify cancer-specific alterations which could then be detected with PCR primers specific for those alterations (again focusing on cancer breakpoints which can be readily observed from limited mate-pair sequencing). PCR primers produced could then be used with digital droplet PCR to quantitate the amount of circulating tumor-free DNA from the blood of that patient which was obtained just prior to surgery. This will quantify the amount of tumor prior to surgery. Then, blood obtained from those patients several days to a week post-surgery could be used to determine the effectiveness of that surgery to eradicate all, or most, of the tumor. For those patients where there is almost no residual cancer DNA, it may not be necessary to follow-up with chemotherapy or radiotherapy and those patients could be monitored with blood draws every six months to determine if there is any cancer recurrence. For those patients that do have residual tumor DNA detected, they could then proceed to subsequent clinical treatments which could also be monitored with the liquid biopsy to determine their effectiveness. This could therefore prove to be a powerful clinical tool for monitoring patients through their treatments.

## 10. Mate-Pair Sequencing As a Powerful Clinical Tool for Cancers with a Viral Etiology

In conclusion the power of limited sequencing of mate-pair libraries produced from DNA isolated from tumors with a viral etiology can already be a very useful tool to aid clinicians in evaluating and treating these patients. For patients with cervical cancer the ability of this approach to detect all the sites of viral integration could help to differentiate patients with potentially less aggressive cancers from those with more aggressive cancers (for example the cervical cancer that killed Henrietta Lacks). For patients with OPSCC this approach can determine which patients have viral integrations and where those integrations occur. Thus, this could also identify patients whose tumors might be more aggressive based upon the sites of integration. For the majority of OPSCC patients, who will not have viral integrations, this approach will still determine the copy number of episomal HPV sequences present which could also have clinical significance. It is, at present, unclear for the other anogenital cancers with an HPV etiology what role HPV plays in these cancers, but this will soon be addressed using next generation sequencing technologies. 

There are other DNA viruses that an involved in the development of cancer, such as hepatitis B in hepatocellular carcinoma. HBV tends to have many more viral integrations in each hepatocellular carcinoma than HPV does in cervical cancer [92], but this information could still help to determine, based upon the genes at or near these integrations, which cancers might be more aggressive. Further studies with other viral-associated cancers using mate-pair sequencing could help to determine the relationship between the physical status of those viruses within an individual cancer and its clinical significance.

In addition to determining the physical status of viruses in each cancer, limited mate-pair sequencing can also provide a high resolution cytogenetic characterization of each cancer. Thus, it can identify regions of chromosomal amplification and regions of deletions. Further studies correlating different specific alterations with clinical outcome could reveal a number of these alterations that do have clinical significance, thus, in the future, this could provide very important information about each specific cancer and its potential to be more or less aggressive.

Finally, and perhaps most powerful of all, the partial sequencing of mate-pair libraries can identify alterations present in each cancer. In our work with mate-pair sequencing of OPSCCs we have found that each OPSCC has multiple alterations that are readily detected. This could include human-HPV junctions in those cancers that do have integration, but also includes several inter-chromosomal translocations and multiple intra-chromosomal alterations as a result of either insertions or deletions. These alterations are cancer-specific (as we also did mate-pair sequencing of DNA isolated from matched normal tissue from each patient) thus are powerful markers for the detection of circulating tumor-free DNA. Then the power of digital droplet PCR to determine the concentration of this specific tumor DNA in the blood from those patients provides a powerful liquid biopsy to monitor the clinical course of that cancer in that patient. This will quickly transform how clinicians can reliably and accurately follow the clinical treatment of their patients.
